# Perturbed autophagy intervenes systemic lupus erythematosus by active ingredients of traditional Chinese medicine

**DOI:** 10.3389/fphar.2022.1053602

**Published:** 2023-01-17

**Authors:** Rui Tian, Lin Yuan, Yuan Huang, Rui Zhang, Hao Lyu, Shuai Xiao, Dong Guo, Declan William Ali, Marek Michalak, Xing-Zhen Chen, Cefan Zhou, Jingfeng Tang

**Affiliations:** ^1^ National “111’’ Center for Cellular Regulation and Molecular Pharmaceutics, Key Laboratory of Fermentation Engineering (Ministry of Education), Cooperative Innovation Center of Industrial Fermentation (Ministry of Education and Hubei Province), Hubei Key Laboratory of Industrial Microbiology, Hubei University of Technology, Wuhan, China; ^2^ Membrane Protein Disease Research Group, Department of Physiology, Faculty of Medicine and Dentistry, University of Alberta, Edmonton, AB, Canada; ^3^ College of Biological Science and Technology, Hubei MinZu University, Enshi, China; ^4^ Hubei Provincial Key Laboratory of Occurrence and Intervention of Rheumatic Diseases, Enshi, China; ^5^ Department of Biological Sciences, University of Alberta, Edmonton, AB, Canada; ^6^ Department of Biochemistry, University of Alberta, Edmonton, AB, Canada; ^7^ Lead Contact, Wuhan, China

**Keywords:** traditional Chinese medicine, systemic lupus erythematosus, autophagy, innate immunity, adaptive immunity

## Abstract

Systemic lupus erythematosus (SLE) is a common multisystem, multiorgan heterozygous autoimmune disease. The main pathological features of the disease are autoantibody production and immune complex deposition. Autophagy is an important mechanism to maintain cell homeostasis. Autophagy functional abnormalities lead to the accumulation of apoptosis and induce the autoantibodies that result in immune disorders. Therefore, improving autophagy may alleviate the development of SLE. For SLE, glucocorticoids or immunosuppressive agents are commonly used in clinical treatment, but long-term use of these drugs causes serious side effects in humans. Immunosuppressive agents are expensive. Traditional Chinese medicines (TCMs) are widely used for immune diseases due to their low toxicity and few side effects. Many recent studies found that TCM and its active ingredients affected the pathological development of SLE by regulating autophagy. This article explains how autophagy interferes with immune system homeostasis and participates in the occurrence and development of SLE. It also summarizes several studies on TCM-regulated autophagy intervention in SLE to generate new ideas for basic research, the development of novel medications, and the clinical treatment of SLE.

## 1 Introduction

Systemic lupus erythematosus (SLE) is an autoimmune disease (Marianthi Kiriakidou and Cathy Lee Ching, 2020) that tends to occur in young women (Zhang et al., 2019a). It is primarily characterized by abnormal T cells, overactive B cells, and the production of large amounts of autoantibodies that affect various organs and threaten human life (Tsokos et al., 2016). The clinical manifestations of SLE are often serious and often include inflammation of the kidney, lung and vascular system and central nervous system injury (Qi et al., 2019). Genetic, environmental, and hormonal factors contribute to the occurrence of SLE (Perl, 2009a; Parks et al., 2017; Stojan and Petri, 2018). However, the etiologies of SLE is not certain.

Autophagy is an adaptive metabolic pathway that commonly occurs in eukaryotic cells, and it is highly dependent on lysosomes, which eliminate damaged and aging organelles and biological macromolecules (Levine and Klionsky, 2004). Autophagy is also involved in the delivery of antigens to MHC compartments, lymphocyte survival/homeostasis regulation, and cytokine production (Levine and Deretic, 2007; Virgin and Levine, 2009; Levine et al., 2011). Therefore, autophagy is involved in most aspects of immunity. Autophagy dysregulation is involved in the occurrence and development of SLE. Many studies showed that natural product extracts and their derivatives were effective treatments for SLE. The present review outlined the related concepts and the link between autophagy and SLE and summarized the current status of TCM ingredients that modulate autophagy in the treatment of SLE.

We searched PubMed, Science Citation Index-Expanded (SCIE) database, SpringerLink, and Chinese National Knowledge Infrastructure (CNKI) for domestic and foreign studies. The keyword “autophagy”, “systemic lupus erythematosus”, “immunity”, “active ingredients of traditional Chinese medicine”, and other keywords were used to search for relevant studies.

## 2 SLE

SLE is an autoimmune disease involving multiple systems and organs, including the kidneys, heart, blood vessels, central nervous system, skin, lungs, muscles, and joints, with an incidence of approximately 0.3–31.5 per 100,000 inhabitants per year. The male-to-female ratio is 1:9 (Fanouriakis et al., 2021), and approximately 15% of patients are children under the age of 18 years (Charras et al., 2021). Females represent 90% of SLE cases (Furst et al., 2013), and marked racial/ethnic disparities are very common. Genetic factors, the environment and lifestyle also induce SLE. However, the mechanism and contribution of these factors induce SLE are the focus of current research, but its detailed pathogenesis has not been elucidated (Cross et al., 2014; Barbhaiya and Costenbader, 2016; Woo et al., 2022).

The disease is characterized by innate and adaptive immunity disorders and the abnormal production of autoantibodies. The etiology of SLE generally includes genetic and environmental factors. Individual genetic risk factors may account for only one-third of the heritability observed in individuals with a family history of SLE. A substantial portion of the remaining risk may be attributable to environmental exposures and gene‒environment interactions. Environmental factors include chemical and physical factors, such as dust, heavy metals, organic pollutants, chemicals, smoking, and UV exposure, and lifestyle factors include diet, drinking and sleep quality. Substantial SLE clustering has been observed in family units with a history of SLE or related autoimmune disease (Dedrick et al., 2020). An 86-fold increased relative risk of SLE was observed in identical twins, with an estimated heritability between 44% and 66% (Ulff-Moller et al., 2018). Genome-wide association studies estimate that genetic risk factors account for only approximately 30% of the observed heritability (Morris et al., 2016). This result suggests that environment and environment-gene interactions are key contributors to the induction of SLE (Ulff-Moller et al., 2018). Approximately 60% of SLE risk may be attributable to environmental exposures (Kuo et al., 2015). The risk in many cases may be due to an environmental triggering of epigenetic modifications that favor differential gene expression. This hypothesis is supported by the fact that in two people with similar genetic risk factors, one person developed SLE due to environmental triggers), and the other person did not develop SLE.

The development of SLE is closely related to sex hormones, especially estrogen. Estrogen levels are significantly higher in patients with active SLE than inactive SLE and significantly higher than normal (Costenbader et al., 2007; Roach et al., 2015). Estrogen controls development, homeostasis, gene expression, and signaling processes in T and B lymphocytes, which influences their function in health and disease (Moulton, 2018). The importance of sex hormone disorders in the pathogenesis of SLE has received much attention over the years. Therefore, understanding the role of estrogen in the pathogenesis of SLE and the efficacy of TCMs associated with estrogen in SLE patients is important (Xiaolan, 2009). Estrogen receptor α/β (ERα/ERβ) balance regulates the activation and proliferation of immune cells and the secretion of cytokines, which play a role in the development and progression of SLE (Moulton, 2018). Based on the pathological mechanism of ERα/ERβ balance in SLE, most TCM clinics use detoxification, elimination of blood stasis and nourishment of the kidney to adjust ERα/ERβ balance as the basic therapy for SLE (Zhou et al., 2015a).

Despite the relatively high heritability of SLE in related individuals, no important susceptibility gene has been identified in the clinic (Mathias and Stohl, 2020). Most of the known SLE susceptibility genes (or genotypes) are not common or sufficiently specific to be used for diagnosis. This factor hinders the early diagnosis and screening for SLE. Patients are often grouped according to known SLE-associated variants with potential roles in SLE pathogenesis, particularly with innate immunity, cellular homeostasis, and adaptive immunity (Teruel and Alarcon-Riquelme, 2016; Teruel et al., 2017).

## 3 Autophagy

The word “autophagy” originally comes from the Greek word meaning “self-eating” (Rout et al., 2014), which is known as type-II cell death (Rouschop and Wouters, 2009). Recent studies rediscovered the physiological phenomenon of autophagy and showed that the process of autophagy had important physiological significance (Singh et al., 2009; Wu and Adamopoulos, 2017; Li and Chen, 2019; Lin et al., 2021). Autophagy at basal levels contributes to the physiological turnover of proteins and the clearance of some old or damaged organelles (Levine and Kroemer, 2019). Various factors, such as lack of trophic factors, hypoxia, accumulation of protein aggregates, and bacterial and viral infections, increase the levels of autophagy (Zhou and Zhang, 2012). Therefore, any changes in the process of autophagy may affect the normal metabolism of cells and result in cell dysfunction. Recent studies showed that autophagy played a very important role in the development, differentiation and maturation of the immune system (Barbhaiya and Costenbader, 2016; Woo et al., 2022). Autophagy also occurs in autoimmune diseases due to impaired immune tolerance. Autoimmune disorders damage organs, such as the pancreas in type 1 diabetes, and systemic lupus erythematosus, which damages tissues and organs throughout the body (Clarke et al., 2015).

**FIGURE 1:**
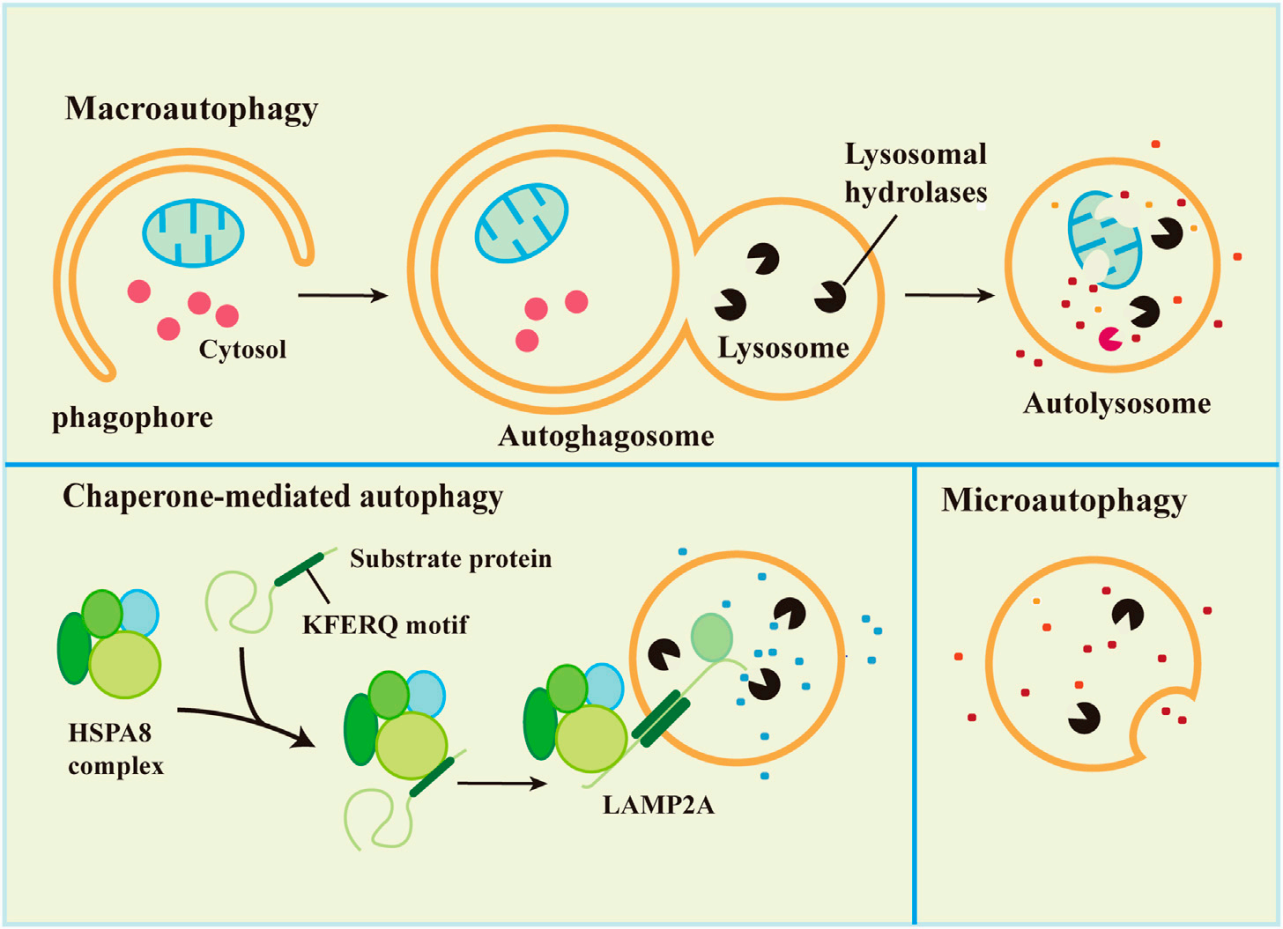
Types of autophagy in mammalian cells.

Synthesis and degradation are the most important processes in maintaining balance in the body. There are two main protease degradation systems in organisms, the ubiquitin-proteasome degradation pathway (Ulff-Moller et al., 2018) and the autophagy-lysosomal degradation pathway. According to the mode of delivery of cytoplasmic material to lysosomes, autophagy has three common forms, macroautophagy, microautophagy, and chaperone-mediated autophagy (CMA), which differ in the mode of cargo delivery to the lysosome (Galluzzi et al., 2017), as shown in Figure 1. Microautophagy, macroautophagy and chaperone-mediated autophagy are coordinated and complementary processes (Li et al., 2012).

### 3.1 Macroautophagy

Macroautophagy is the most studied autophagy pathway. Macroautophagy (hereafter referred to as autophagy) is the process of degrading cytoplasmic components by isolating and encapsulating cytoplasm using unique bilayer vesicles (autophagosomes) that fuse with lysosomes to form autophagic lysosomes (Glick et al., 2010). Macroautophagy is characterized by the appearance of newly synthesized autophagosomes, fusion with lysosomes, and the degradation of lysosomes (Levine et al., 2011). Autophagy includes 5 key processes: 1) formation of the phagophore, 2) Atg5-Atg12-Atg16L complex formation and fusion with the phagophore, 3) (microtubule-associated protein light chain 3 (LC3) transformation from a soluble form (LC3-I) to a lipid-soluble form (LC3-II) and binding with the phagophore to form autophagosomes, 4) autophagosome capture of proteins, organelles and other substances for degradation or removal, and 5) autolysosome formation *via* the combination of autophagosome and lysosome followed by degradation of the content in the autophagic lysozyme membrane (Wu and Adamopoulos, 2017). The process is shown in Figure 1.

### 3.2 Microautophagy

Microautophagy is classified into two types according to the molecular mechanism of cargo uptake: fission-type and fusion-type microautophagy (Schuck, 2020). Microautophagy is the phagocytosis of substrate molecules in the cytoplasm *via* invagination of the lysosomal membrane. The substrate of microautophagy is directly involved in the structure of microtubule vesicles by a lysosomal membrane, which are phagocytosed instead of degraded by the formation of autophagosomes (Mijaljica et al., 2011). Microautophagy primarily includes 5 processes: 1) microautophagy invagination and formation of autophagic tubes, 2) vesicle formation, 3) vesicle expansion, 4) vesicle rupture, and 5) vesicle degradation (Li et al., 2012).

Microautophagy is inherent in mammalian cells (Zhou et al., 2015a). Similar to macroautophagy, starvation and rapamycin induce microautophagy, which may be due to the absence of rapamycin target proteins or starvation-induced autophagy in macrophages (Li et al., 2012). Microautophagy plays an important role in the maintenance of membrane homeostasis. The membrane consumption rate of microautophagy must be equal to the membrane influx rate of macroautophagy for membrane homeostasis (Todde et al., 2009). Most autophagosomes are derived from microautophagy, and microautophagy and macroautophagy help cells withstand starvation to maintain physiological functions *via* continuous nutrient and energy circulation (Muller et al., 2000; Dalby et al., 2010). Microautophagy plays an important role by regulating the ratio of lipids-to-proteins on the surface of lysosomes (Iwata et al., 2005). Membrane proteins may be renewed by microautophagy in endosome chaperone-mediated autophagy (Saksena and Emr, 2009) or used as an energy transfer pathway (Takikita et al., 2009).

### 3.3 Chaperone-mediated autophagy

The main feature of CMA is that neither vesicles nor membrane invaginations are required for substrate delivery to lysosomes. Substrates reach the lysosomal lumen *via* a protein-translocation complex at the lysosomal membrane. The substrate protein binds to the molecular chaperone and is transported directly into the lysosome (Zhou and Zhang, 2014; Galluzzi et al., 2017). Chaperone-mediated autophagy is a unique and selective autophagy that degrades proteins with KFERQ sequences in the cytoplasm *via* the lysosomal-protein pathway.

### 3.4 Commonly used methods for autophagy monitoring

Electron microscopy, detection and quantification of Atg8 family proteins, SQSTM1 and related LC3-binding protein conversion analysis are commonly used methods for monitoring autophagy. Electron microscopy observes autophagic vesicles, and TEM (Cardenal-Munoz et al., 2017) tracks sequential morphological changes during autophagy. The maturation of phagosomes *via* autophagic lysosomes is a dynamic and continuous process (Eskelinen, 2005). Atg8 is a ubiquitin-like protein. Atg8 exists in the conjugated form of Atg8-PE and possibly to phosphatidylserine in yeast and some other organisms (Sou et al., 2006). Atg8 and Atg8 family proteins are the most widely monitored autophagy-related proteins. SQSTM1 protein acts as a link between LC3 and ubiquitinated substrates, and SQSTM1 protein and its bound polyubiquitinated proteins are integrated into intact autophagic vesicles and degraded in autophagic lysosomes, which supports its use as an indicator of autophagic degradation (Bjorkoy et al., 2005).

## 4 Autophagy and immunological aberrations in SLE

**FIGURE 2:**
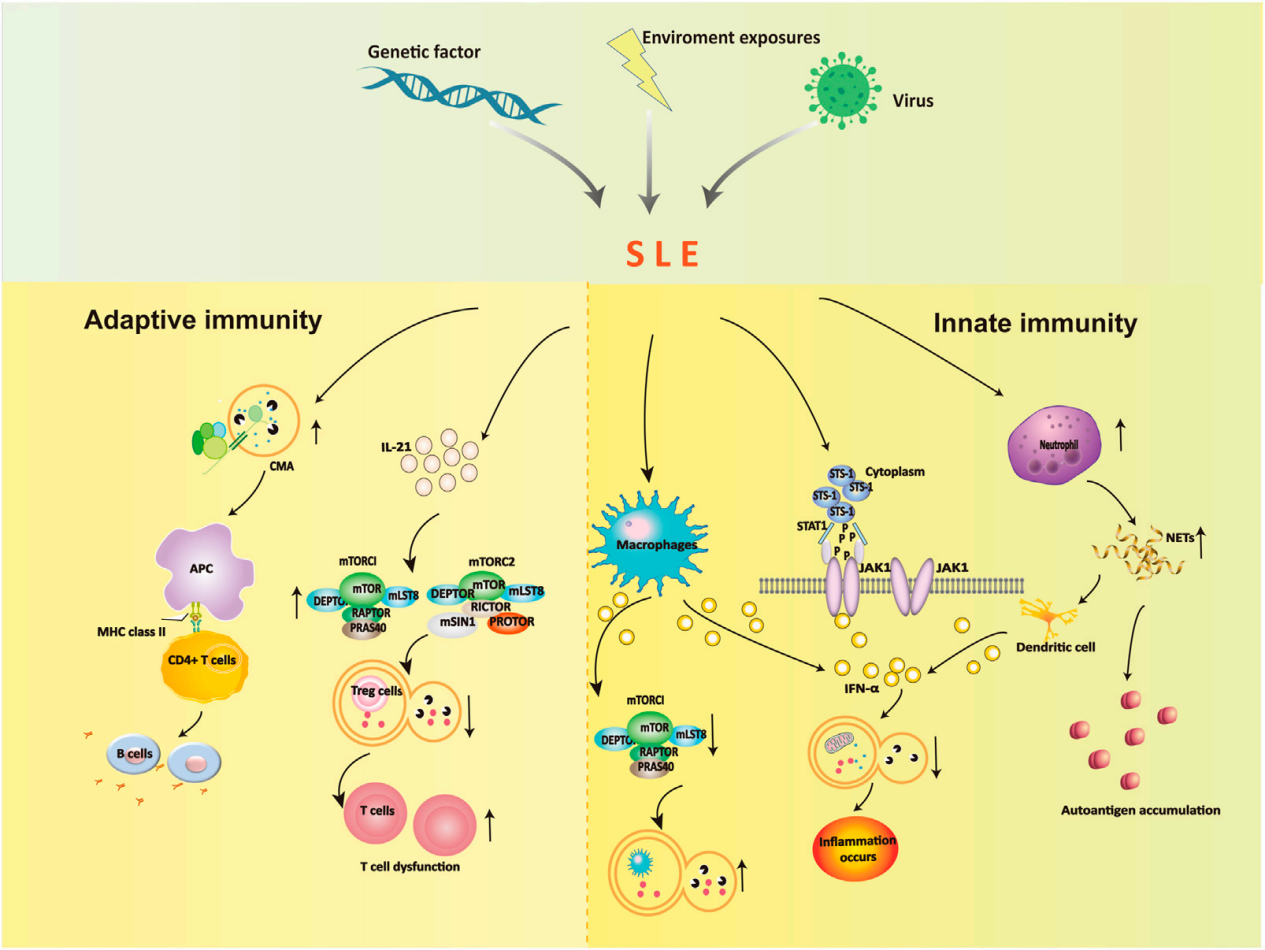
The role of autophagy in the dysregulation of innate immunity and adaptive immunity.

Autophagy is a programmed intracellular degradation mechanism. An increased number of autophagosomes are observed in many diseases, including autoimmune diseases, tumors, infections, and cardiovascular and cerebrovascular diseases (Choi et al., 2013; Deretic et al., 2013). The pathogenesis of SLE is likely based on genetic susceptibility factors of the body due to infection, ultraviolet (UV) irradiation, and other factors, inducing immune disorders that result in an abnormal activation of autoreactive T and B lymphocytes, the production of a large number of autoantibodies, immune complex formation and deposition, which lead to deregulated inflammation and multiorgan injuries (George and Tsokos, 2011; Lech and Anders, 2013; Marianthi Kiriakidou and Cathy Lee Ching, 2020). Jian Zheng (Zheng et al., 2022)analyzed the B and T cell subsets comparing SLE patients in the low activity phase and healthy controls, and the T and B cell axes showed abnormalities, and the proportion of double negative B cells and CD8^+^ T cells was significantly reduced, the results indicated that the immune phenotype and the incidence of the disease were closely related. Autophagy affects the pathogenesis of SLE in many ways. Autophagy is recognized as a central pathogenic factor in immune abnormalities of SLE, and it affects innate and adaptive immunity (Harley et al., 2008; Han et al., 2009). The immune system is responsible for surveillance and communication between different organs and cell types, and the role of autophagy and the consequences of autophagy deficiency go far beyond the degradation of this pathway (Deretic, 2012; Bhattacharya and Eissa, 2013). Autophagy is involved in many aspects of SLE, including the clearance of dead cells, intracellular DNA and RNA, regulation of the response to type Ⅰ interferon (IFN), and control of the long-term survival of B and T cells (Rönnblom and Pascual, 2008; Clarke et al., 2015; Bhattacharya et al., 2015). Recent studies showed that defects in macroautophagy/autophagy contributed to the pathogenesis of SLE, especially in adaptive immunity (Zhou et al., 2019). The link between environmental stimuli and autophagy in SLE is indirect, but we can speculate about their relationship (Figure 2).

### 4.1 Autophagy and innate immunity in SLE

The effects of autophagy on the occurrence and development of SLE are partially due to the influence of innate immune dysregulation. Autophagy plays a role is the elimination of pathogens in innate immunity. Autophagy controls pathogen recognition and intracellular killing. Macrophages are professional phagocytic cells in the body that engulf pathogens, foreign bodies, and dead cells. Autophagy is necessary for the removal of dead cells. Apoptotic cells release lysophosphatidylcholine (LPC) as a marker signal to induce phagocytic cells and upregulate phosphatidylserine (PS) as a phagocytosed signal on their surface. The efficient release of LPC is critical for autophagy genes, and apoptotic cells cannot normally express PS on their surface in the absence of autophagy (Qu et al., 2007). The main mechanism in SLE patients is abnormal immune type I IFN secretion by macrophages. High expression of IFN-α inhibits mTORC1 and activates reactive oxygen species to induce autophagy in podocytes, which leads to microtubule-associated protein 1 light chain 3 accumulation and a decrease in p62 (Dong et al., 2015). These alterations block the degradation of mitochondrial components, such as autophagic degradation of mitochondrial DNA, and lead to inflammation.

Neutrophils prevent infection in the body and are the most important phagocytic cells. Increased expression of neutrophil-specific transcripts correlates with the development of nephritic disease (Bennett et al., 2003). Neutrophils immobilize and kill invading microorganisms by forming a neutrophil extracellular trap. Neutrophils exhibit an enhanced ability to form neutrophil extracellular traps (NETosis) containing self-antigens in SLE patients, including chromatin, dsDNA, and granulin. NETosis is increased in SLE, which results in an excess load of nuclear autoantibodies (Pieterse and van der Vlag, 2014). The clearance of NETs is diminished in SLE patients, and dendritic cells are stimulated to produce type I interferons, which contribute to the pathogenesis of SLE (Garcia-Romo et al., 2011).

Another potential factor is the suppressor of T-cell receptor signaling 1 (STS-1), which promotes IFN-α-activated JAK1-STAT1 signaling *via* c-cbl dephosphorylation, inhibits the PI3K-mTOR signaling pathway and activates autophagy (Dong et al., 2015). The protein tyrosine phosphatase STS-1 is significantly overexpressed in B cells in SLE patients and MRL/lpr mice (Dong et al., 2015). Notably, STS-1 inhibits autophagy *via* JAK1-STAT1 and activates the autophagy pathway *via* the mTOR pathway, which may serve as an important target for the future treatment of SLE.

### 4.2 Autophagy and adaptive immunity in SLE

#### 4.2.1 B cells

The dysregulation of humoral immunity in SLE leads to the abnormal differentiation of B cells. B cells are primarily involved in chaperone-mediated autophagy (CMA), and the occurrence of SLE leads to enhanced autophagy activity, which leads to further abnormal B-cell differentiation that participates in the pathogenesis of SLE. Inhibiting the autophagy of B cells in SLE patients blocked the production of antinuclear antibodies and sharply reduced the secretion of inflammatory factors, which caused the inflammation to disappear (Weindel et al., 2015). Molecular chaperone-mediated autophagy in B cells is involved in the pathogenesis of SLE, and the abnormal level of this autophagy is closely related to the progression of the disease.

Studies showed that (Page et al., 2011; Macri et al., 2015) SLE altered the activity of CMA. The CMA markers LAMP-2A and HSPA8 were overexpressed, in spleenic B cells of lupus-prone MRL/LPR mice, and CMA-associated lysosomes were 1.6-fold higher than non-lupus-prone CBA/J mice. Due to the increase in CMA activity in SLE patients, many autoantigens are processed and loaded onto MHC II molecules and presented to autoreactive CD4^+^ T cells, which promotes autoreactive B cells to proliferate and differentiate into plasma cells. Many harmful autoantibodies are secreted and eventually cause the pathogenesis of SLE.

#### 4.2.2 T cells

Autophagy is regulated by the PI3K/Akt/TSC/mTOR signaling pathway, which is closely associated with SLE (He et al., 2019). The mitochondrial hyperpolarization site is located upstream of mTOR, which promotes mTOR activation and leads to the upregulation of intracellular calcium influx. This influx abnormally activates T lymphocytes and ultimately leads to the occurrence of SLE (Alessandri et al., 2012). One study found a reduced number of CD8^+^ memory T cells in the peripheral blood of SLE patients (Fernandez and Crow, 2018). The number of memory CD8^+^ T cells was increased after treatment with the mTOR inhibitor rapamycin (RAPA). These results showed that the differentiation of naive CD8^+^ T cells was closely associated with the activation of mTOR in the peripheral blood of SLE patients. Therefore, we conclude that mTOR may be involved in the development of SLE. Kato et al. found that IL-21 activated mTORC1 and mTORC2, inhibited autophagy, hindered the differentiation of initial CD4^+^ T cells in the peripheral blood of SLE patients and healthy people to Tregs, and inhibited the function of Tregs. After 4 weeks of treatment with rapamycin, autophagy reappeared, and the function of Tregs returned to normal, which confirmed the role of mTOR in the pathogenesis of SLE *via* autophagy regulation from a qualitative point of view (Kato and Perl, 2018; Crino, 2019). The increase in mTORC1/mTORC2 activity in SLE patients is due to stimulation of the secretion of the inflammatory factor IL-21, which reduces autophagy and blocks the differentiation function of CD4^+^ and CD25^+^ Tregs. One form autophagy removes abnormal mitochondria. Because the reduction in autophagy in T cells leads to mitochondrial dysfunction, the accumulation of a large number of mitochondria will further lead to T-cell dysfunction and aggravation of SLE. Constitutive autophagic damage in T lymphocytes in SLE patients is associated with an abnormal accumulation of α-synuclein aggregates (Colasanti et al., 2014).

Although the exact mechanism leading to dysregulation of autophagy in SLE is not clear, altered levels of autophagy have been implicated in the survival of autoimmune T and B cells. A link between autophagy and SLE has been established. Observations that drugs modulating autophagy, including hydroxychloroquine (Ruiz-Irastorza et al., 2010), rapamycin (Perl, 2009b; Page et al., 2011), and the P140 peptide (Page et al., 2011), provide beneficial effects in lupus mouse models and lupus patients support that modulating autophagy levels may be an important therapeutic target.

### 4.3 Genome-wide association study of SLE

With the advancement of genome-wide association studies (GWAS), the number of established genetic associations with SLE is also increasing. Several studies found that ATG5 (Pierdominici et al., 2012) and ATG7 were closely related to the pathogenesis of SLE (Han et al., 2009; Yang et al., 2010). Another study found significant hypomethylation of differentially methylated sites associated with several interferon-related genes, including MX1, IFI44 L, PARP9, DT3XL, IFIT1, IFI44, RSAD2, PLSCR1, and IRF7, which suggests the importance of the I-IFN pathway in the pathogenesis of SLE (Joseph et al., 2019). Environmental factors stimulate the initiation of autoimmunity in genetically susceptible individuals, and the expansion of its role in autophagy initiation and SLE development may help elucidate the etiological role of autophagy in SLE. These findings provide interesting clues for elucidating the mechanism of autophagy in the pathogenesis of autoimmune diseases, such as SLE, and identify a new layer of biological avenues for future targetable treatments.

Autophagy affects the pathogenesis of SLE *via* many aspects. Therefore, autophagy pathway genes have become a new direction for SLE target therapy. Bortezomib, cyclosporine A, rapamycin, P140 phosphopeptide, vitamin D, and glucocorticoids are related to autophagy (Perl, 2009b; Ruiz-Irastorza et al., 2010; Page et al., 2011; Lai et al., 2013; Harr et al., 2014; Lamanna et al., 2014; Lesovaya et al., 2015; Gu et al., 2016).Glucocorticoids and immunosuppressants are the basic treatment regimens for this disease, but mortality is increased due to complications and infections associated with the long-term use of hormone drugs (Qi et al., 2013). One study showed that combined therapy with Chinese medicine may improve the survival of SLE patients, and traditional Chinese medicine has good safety (Ma et al., 2016).

## 5 Traditional Chinese medicine as an autophagy modulator for SLE treatment

At present, the treatment of SLE with TCM is mainly focused on theoretical research stage, and clinical research is less reported. Previously, the research of TCM for SLE mainly focused on some compound drugs, and with the development of natural medicinal chemistry, the active ingredients of TCM gradually became the focus of research. TCM has become an important field of new drug research and development in the search for drugs that interfere with SLE by regulating autophagy. Pharmacological methods to activate or inhibit autophagy are necessary because autophagy plays a protective role in pathological conditions and at different stages of the same diseases. TCMs are typically categorized as alkaloids, flavonoids, saccharides, saponins, terpenoids, and polyphenols based on their chemical properties (Table 1).

**TABLE 1:** Novel treatment strategies targeting SLE *via* autophagy.

Category	Compounds	Herbs	Function	References
Glucosides	Total glucosides of paeony	The roots of *Paeonia lactiflora*	The expression rates of CD4^+^, CD25^+^T cells in SLE patients were significantly increased	(Lai et al., 2013; Gu et al., 2016)
	Glycyrrhizin	*Glycyrrhiza uralensis* Fisch	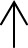 Autophagy increased the expression level of CD4^+^ and CD25+T cells	(Shang-shang, 2015; Li et al., 2019)
Polysaccharide	Pachyman polysaccharides	*Poria cocos (Schw.)*Wolf	Regulated the balance of Th17/Treg	Pastorino et al. (2018)
	*Astragalus* polysaccharides	*Astragalus membranaceus*(Fisch.)Bye	Regulated T-cell subsets to return to normal	(Kim et al., 2012; Xu et al., 2020)
Flavonoids	Micromeraceae. Icaritin	*Epimedium brevicornum* Maxim.	Regulated autophagy level and inhibited T-cell overactivation	Yarley et al. (2021)
	Dihydromyricetin	*Ampelopsis megalophylla* Diels et Gilg	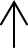 Autophagy inhibited the activity of mTOR	Talaat et al. (2015)
Terpenoids	Triptolide	the root of *Tripterygium wilfordii*	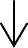 Autophagy	Alunno et al. (2012)
			Inhibited the JAK/STAT signaling pathway	Tang et al. (2021)
	Artemisinin	*Artemisia annua* L.	Regulated autophagy	Zhang et al. (2018)
Others	Resveratrol	*Polygonum cuspidatum*	Inhibited the proliferation of B lymphocytes and the activation of CD4^+^ T cells	Wu et al. (2018)
	Curcumin	*Curcuma longa* L.	Enhanced the function and number of Treg cells	Jacqueline et al. (2021)
	Embelin	*Embelia ribes* Burm. f.	Regulated the balance of Th-cell subpopulations and inhibited the excessive activation of Th and B cells	Han et al. (2009)

### 5.1 Glucosides

Glycosides are sugars or derivatives of sugar compounds that are linked to another type of monosaccharide by an anomeric carbon atom of the sugar. Total glucosides of peony (TGP) are glycosides extracted from the roots of *Paeonia lactiflora*, a plant of the Ranunculaceae family (Li et al., 2019). TGP inhibits CD11a gene expression by enhancing the DNA methylation of a promoter in CD4^+^ T cells, CD11 (Shang-shang, 2015), and autoimmunity by inducing Treg cell differentiation (Zhao et al., 2012). There is a correlation between the course of SLE and the level of CD4^+^ and CD25^+^ T cells (Sun et al., 2008). The expression rate of CD4^+^ and CD25^+^ T cells in active SLE patients was significantly lower than healthy control group. The expression rates of CD4^+^ and CD25^+^ T cells in SLE patients were significantly increased after treatment. TGP therapy may act on CD4^+^ and CD25^+^ T cells (Yiping et al., 2014). These results provide evidence for the mechanism of action of TGP in the treatment of SLE.

Licorice is a Chinese herb tonic that exhibits anti-inflammatory, antibacterial, antiviral, and antitumor effects (Sun et al., 2021). The compound glycyrrhizin is the main raw material, and it contains glycyrrhizin, glycine, cysteine, glycyrrhizin, and other components. The combination of compound glycyrrhizin and prednisone had a good effect on SLE (Kim et al., 2012; Pastorino et al., 2018; Sun et al., 2021). Autophagy promotes the survival of T lymphocytes and plays an important role in inducing apoptosis during T lymphocyte proliferation (Xu et al., 2020). Autophagy was observed at different stages of T-cell activation in patients with SLE (Zhou et al., 2015b). CD4^+^, CD25^+^Treg cells are a subset of regulatory T cells that maintain autoimmune tolerance and regulate the immune response. Dysfunction or a decrease in the number of CD4^+^, CD25^+^ Treg cells is an important cause of autoimmune diseases (Xu et al., 2020). Compound glycyrrhizin with prednisone more effectively increased the expression levels of CD4^+^, CD25^+^ T cells to regulate the immune state of SLE patients and achieve immune balance (Li and Yang, 2011).

### 5.2 Polysaccharides

A polysaccharide is a sugar composed of more than 10 monosaccharides connected by a glycosidic bond. Polysaccharides produce a variety of pharmacological effects, and its biological activity is closely related to the type of glycosidic bond, degree of branching, and functional groups (Zhang et al., 2019b; Yarley et al., 2021). Polysaccharides have immunomodulatory and anti-inflammatory activities (Li et al., 2018). A significant imbalance of Th17/Treg was observed in the pathogenesis of SLE, which suggests that Th17/Treg affect the occurrence and development of SLE (Talaat et al., 2015). Pachyman polysaccharides regulate the balance of Th17/Treg by reducing Th17 and increasing Treg cells, which provides a new strategy and entry point for the treatment of SLE (Wang et al., 2017). *Astragalus membranaceus* is the root of the legumes *Astragalus* mongolicus and *Astragalus* membranaceus. It has many functions, such as anti-inflammatory, hypoglycemic, and lipid-lowering activities, and contains a variety of active ingredients, such as flavonoids, polysaccharides, amino acids, alkaloids, and linoleic acid. *Astragalus* polysaccharides (APS) have the strongest immune activity (Jiang et al., 2020). APS returned T-cell subsets to normal in patients with SLE (Wei et al., 2021). Autophagy promotes the survival of T lymphocytes and plays an important role in inducing apoptosis during T lymphocyte proliferation (Zhou et al., 2015b). Therefore, the activation of autophagy and regulation of T-cell homeostasis may be the main mechanism of polysaccharide compounds in the treatment of SLE.

### 5.3 Flavonoids

Flavonoids are a series of compounds formed by the connection of two benzene rings with phenolic hydroxyl groups *via* the central three carbon atoms. A large number of studies demonstrated that cell and cell imbalance were closely related to the occurrence and development of autoimmune diseases, especially SLE (Alunno et al., 2012). The development and proliferation of T lymphocytes depend on autophagy (Alunno et al., 2012). The flavonoid icaritin (ICT) is the metabolic product of icarioside. ICT inhibited T-cell overactivation in SLE patients (Yue, 2014). Therefore, ICT may improve SLE patients by regulating autophagy levels and inhibiting T-cell overactivation.

Dihydromyricetin (DMY or DHM), also known as ampelopsin, dihydromyricetin, and fukiencha, is a dihydroflavonol flavonoid compound (Zhang et al., 2018). Dihydromyricetin promoted the expression of LC3-II and Beclin-1 autophagy genes in a dose- and time-dependent manner and inhibited the activity of mTOR (Xia et al., 2014). Autophagy is regulated by the PI3K/AKT/mTOR signaling pathway, which is closely related to SLE (Jin et al., 2016). Chen Yanwen et al. (Tao and Qing-Jun, 2015) found that the autophagy level of T lymphocytes in SLE patients was higher than normal subjects, and the autophagy level in the active group of SLE patients was significantly higher than the stable group. The expression of a marker related to autophagy, mTOR, negatively correlated with the disease condition. Therefore, regulating autophagy *via* the mTOR signaling pathway may be the main target of flavonoids in the treatment of SLE, but the specific link and target must be further studied and clarified. There are few studies on the mechanism of flavonoid action on SLE, and further studies are guiding the development of flavonoids into new Chinese medicine drugs for the prevention and treatment of SLE.

### 5.4 Terpenoids

Terpenoids are the most abundant compounds in natural products and are derived from the root of *Tripterygium wilfordii* (Li et al., 2014). Terpenoids have many biological activities. Triptolide (TP) is an epoxide diterpene lactone compound that has strong anti-inflammatory and antitumor effects. TLR7 agonists (R848 and imiquimod) induced excessive autophagy in RAW264.7 cells *in vitro* and promoted the expression of anti-dsDNA and the secretion of some inflammatory IgM and IgG cytokines *in vivo*, which produced symptoms of SLE disease (Yokogawa et al., 2014; Li et al., 2016). Using SLE mice as a model, the levels of inflammatory factors (IL-6, IL-10, TNF-α, and IFN-γ) were reduced in the triptolide group compared to the model group, and the expression levels of JAK, p-JAK1, STAT3, and p-STAT3 in renal tissues were significantly reduced (*p* < 0.05). These results indicated that triptolide inhibited the JAK/STAT signaling pathway, alleviated the inflammatory immune response and improved the condition of SLE in mice (Tang et al., 2021). Wu et al. (Wu et al., 2018) found that R848 induced excessive autophagy in RAW264.7 cells to form an SLE model *in vitro*, and the expression of the autophagy-related proteins LC3II/I and P62 was significantly decreased with TP intervention. These results suggested that triptolide regulated the expression of autophagy proteins, and its effects were related to the PI3K/AKT/mTOR signaling pathway.

Artemisinin is an active ingredient extracted from *Artemisia annua* L. and is a sesquiterpene lactone (Feng et al., 2021). Artemisinin and its derivatives have good anti-inflammatory immunosuppressive effects. Artemisinin exerts anti-inflammatory effects primarily by enhancing the level of autophagy (Qian et al., 2017; Qi et al., 2019). Dihydroartemisinin significantly upregulated the protein expression levels of LC3-II and ATG5. The mTOR and ULK1 signaling pathways regulate the generation of autophagosomes (Kang et al., 2015). The effects of terpenoids on SLE involve many mechanisms and may include the regulation of autophagy. However, the specific targets of terpenoids must be further explored.

### 5.5 Other agents

Resveratrol is a polyphenolic compound derived from various plants that has antioxidant, antitumor, and anti-inflammatory effects, and it may be used to treat cardiovascular diseases (Wang et al., 2006; Soylemez et al., 2008; Ingmer and Ingmer, 2019). Resveratrol regulates autophagy *via* a variety of pathways (Jacqueline et al., 2021; Li et al., 2021; Sha et al., 2021). Autophagy plays an important role in the activation and proliferation of B cells. Activation of autophagy is the survival mechanism of autoreactive B cells. It plays an important role in the differentiation of B cells to plasma cells and the humoral response and provided metabolic support for proliferative lymphocytes (Bernard, 2014; Clarke et al., 2015). Resveratrol inhibits the proliferation of B lymphocytes and the activation of CD4^+^ T cells (Nakata et al., 2012) and the activation, proliferation, immunoglobulin and proinflammatory cytokine secretion of lupus B lymphocytes and differentiation into plasma cells *in vitro* (Wang, 2012). The adaptive immune response is a key feature of SLE, which leads to dysfunctional T cells and the abnormal activation of B cells, and these functional alterations are involved in the development and progression of SLE. In summary, resveratrol may be a new drug for SLE treatment by regulating autophagy in the future. One recent study showed that Th17 and Treg cells in the peripheral blood of SLE patients were upregulated by autophagy, which was associated with the increased proinflammatory response of Th17 cells and decreased immunosuppression of Treg cells (Xue et al., 2017; Kato and Perl, 2018). Treg cells and their regulatory cytokines are important mediators of autotolerance (Jia Nie et al., 2015). Curcumin is a compound of polyphenols that reduces the Th17-cell response by inhibiting cell proliferation and related inflammatory cytokines and transcription factors. Curcumin may become a natural compound to regulate inflammation in autoimmune diseases, such as SLE, by increasing the secretion of cytokines and enhancing the function and number of Treg cells (Fu et al., 2020). Embelin is a benzoquinone compound extracted from the fruit of Peperomia sylvestre. A study with an SLE mouse model found that the Th1/Th2 and Treg/Th17 ratios were significantly higher in the embelin group compared to the control group (*p* < 0.05), and the expression levels of CD69, CD86, and MHC-II on B cells, CD69 on Th cells, and CD154 expression levels on B cells were significantly lower (*p* < 0.05). Embelin exerted its therapeutic effect in SLE mice by regulating the balance of Th-cell subpopulations and inhibiting the excessive activation of Th and B cells (Shen et al., 2022). Because autophagy is strongly implicated in immune functions, such as the removal of intracellular bacteria, inflammatory cytokine secretion, antigen presentation, and lymphocyte development (Wu and Adamopoulos, 2017), these compounds may exert therapeutic effects in SLE patients by modulating autophagy to regulate immune disorders.

## 6 Discussion and conclusion

SLE is highly heterogeneous, and its pathogenesis has not been fully clarified. There is no effective curative treatment. However, the role of autophagy in the innate and adaptive immunity of SLE is clear because the environment, genetics, and immunity are the chief factors that cause SLE. Under the influence of autophagy genes, autophagy regulates the clearance of apoptotic cells, the type Ⅰ interferon response, and the survival of B cells and T cells, which participate in the occurrence of SLE from many aspects and levels. Autophagy pathway inhibitors reduce patient autoantibodies, immune complexes in the glomerulus, and proteinuria. Therefore, autophagy regulation may provide a new strategy for the treatment of SLE.

The present review summarized some natural compounds derived from herbal medicines that alleviate the progression of SLE by modulating the immune system and directly or indirectly modulating autophagy in SLE patients. Chinese herbal medicine is only used as an adjuvant, and clinical research data on the single application of herbal medicine for disease prevention and treatment are scarce. Current studies lack in-depth exploration of mechanisms, such as the relevant signaling pathways, pathways of action, or specific targets. Exploring the synergistic effects of several compounds with the same or different biological targets is also a frontier for future research. It is important to investigate the mechanisms of autophagy regulation in TCM for the treatment of SLE and identify new therapeutic targets.

Glucocorticoids and immunosuppressants are the treatment regimens and play a therapeutic role in SLE by inhibiting mTOR and activating autophagy. However, the long-term use of hormone drugs leads to some serious toxicity and side effects, especially infection, which increases mortality. The use of TCM combined treatment significantly improves drug safety and the survival rate of patients with SLE. Notably, some types of TCMs cause liver or kidney injury, and the number of reported cases is increasing. Therefore, toxicological investigations must be performed before clinical trials of TCM to determine toxic doses and establish a reasonable standard for the use of TCM.

In conclusion, TCMs certainly regulate SLE disease by promoting or inhibiting autophagy. TCMs consist of complex components that interact with multifunctional targets and pathway. Therefore, the mechanism of TCM treatment of diseases is a complicated interaction network rather than a single pathway. However, the mechanism of cell autophagy regulation is complex and may involve multiple pathways and target proteins, especially due to drug compatibility, dose, or duration, and the same types of effective components may lead to activation or inhibition of the same target protein. Therefore, the material basis of TCM and the regulatory mechanism of autophagy by TCM must also be further investigated and discussed. The regulation of cell autophagy may provide a new strategy for the treatment of SLE.

**FIGURE 3:**
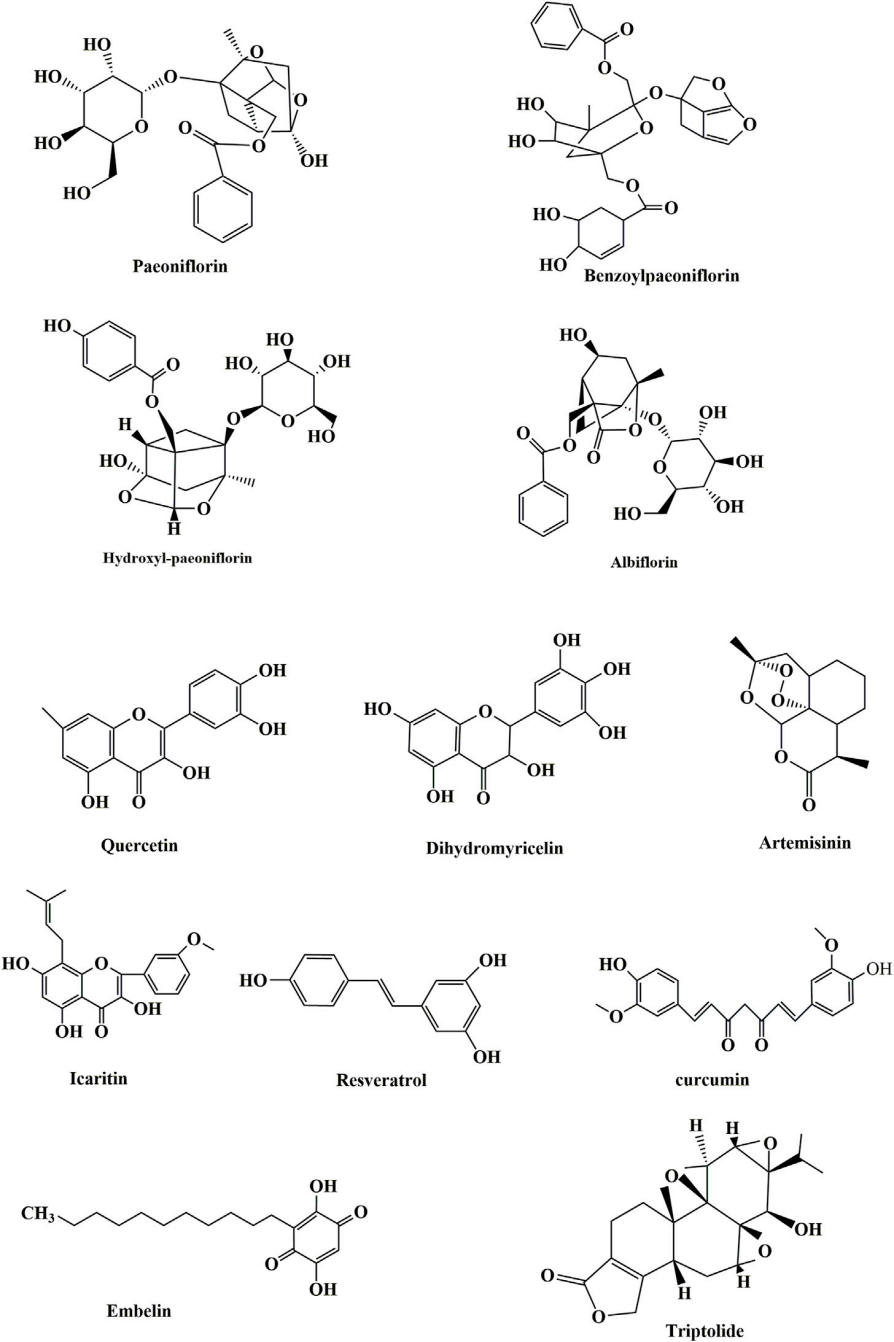
Chemical structures of some compounds.

Research on the role of autophagy in the pathology of SLE, especially in-depth research on the specific mechanism of action, should be further studied. Research on the mechanisms of drug interference with SLE *via* autophagy regulation is superficial, and there is much space for research on the regulation of its specific mechanism of action. The present review outlined the relevant concepts, issues, and conclusions of existing studies. Further extensive studies are needed to elucidate the precise functional role of autophagy in SLE, especially biomarkers and therapy and the molecular mechanism of drugs that interfere with the occurrence and development of SLE *via* autophagy (Figure 3).
